# Overexpression of *Salmonella enterica* serovar Typhi *recA* gene confers fluoroquinolone resistance in *Escherichia coli* DH5α

**DOI:** 10.1590/1414-431X20154804

**Published:** 2015-09-08

**Authors:** M.A.M. Yassien, M.A. Elfaky

**Affiliations:** Department of Natural Products and Alternative Medicine/Microbiology, Faculty of Pharmacy, King Abdulaziz University, Jeddah, Saudi Arabia

**Keywords:** *Salmonella enterica* serovar Typhi, Fluoroquinolones, RecA

## Abstract

A spontaneous fluoroquinolone-resistant mutant (STM1) was isolated from its parent *Salmonella enterica* serovar Typhi (*S. Typhi*) clinical isolate. Unlike its parent isolate, this mutant has selective resistance to fluoroquinolones without any change in its sensitivity to various other antibiotics. DNA gyrase assays revealed that the fluoroquinolone resistance phenotype of the STM1 mutant did not result from alteration of the fluoroquinolone sensitivity of the DNA gyrase isolated from it. To study the mechanism of fluoroquinolone resistance, a genomic library from the STM1 mutant was constructed in *Escherichia coli* DH5α and two recombinant plasmids were obtained. Only one of these plasmids (STM1-A) conferred the selective fluoroquinolone resistance phenotype to *E. coli* DH5α. The chromosomal insert from STM1-A, digested with *Eco*RI and *Hind*III restriction endonucleases, produced two DNA fragments and these were cloned separately into pUC19 thereby generating two new plasmids, STM1-A1 and STM1-A2. Only STM1-A1 conferred the selective fluoroquinolone resistance phenotype to *E. coli* DH5α. Sequence and subcloning analyses of STM1-A1 showed the presence of an intact RecA open reading frame. Unlike that of the wild-type *E. coli* DH5α, protein analysis of a crude STM1-A1 extract showed overexpression of a 40 kDa protein. Western blotting confirmed the 40 kDa protein band to be RecA. When a RecA PCR product was cloned into pGEM-T and introduced into *E. coli* DH5α, the STM1-A11 subclone retained fluoroquinolone resistance. These results suggest that overexpression of RecA causes selective fluoroquinolone resistance in *E. coli* DH5α.

## Introduction

Fluoroquinolones are synthetic antimicrobial agents with a broad spectrum of activity and potent antibacterial activity against Gram-negative bacteria (1). The extensive use of fluoroquinolones for the treatment of typhoid is one important factor that has led to the frequent isolation of fluoroquinolone-resistant isolates in developing countries in Africa and South Asia (2).

Fluoroquinolone resistance in Gram-negative bacteria involves mutations in DNA gyrase that confer various levels of resistance to fluoroquinolones or alterations in drug permeation across the bacterial cell membrane; such mutations can lead to fluoroquinolone resistance and resistance to unrelated classes of antimicrobial agent (3).

Fluoroquinolones are potent inducers of SOS responses in bacteria, and these responses result from induction of more than 20 genes or operons (4). The SOS response plays an important role in repairing the DNA damage caused by, for example, chemical treatments. The RecA protein, a ubiquitous bacterial recombination protein, plays a role in mediating SOS responses in bacteria. Through this response, the RecA protein may play a role in the development of fluoroquinolone resistance (5,6).

A spontaneous fluoroquinolone-resistant mutant (STM1) was randomly isolated from a serovar Typhi clinical isolate of *Salmonella enterica* (*S. Typhi*). The results of DNA gyrase sensitivity testing of this mutant revealed that the development of fluoroquinolone resistance is not the result of mutation in DNA gyrase. The present study focused on identifying the mechanism of fluoroquinolone resistance in the STM1 mutant.

## Material and Methods

### Bacteria and culture media

The *S. Typhi* clinical isolate used herein and characterized by its sensitivity to fluoroquinolones was obtained from the Microbiology Laboratory in the King Abdulaziz University Hospital, Saudi Arabia. The fluoroquinolone-resistant mutant, STM1, was isolated randomly from the parental strain.

The bacterial strains and plasmids used in the present study are listed in Table 1. Unless otherwise noted, bacteria were grown in Luria-Bertani broth (LB, 10 g tryptone, 5 g yeast extract, 10 g NaCl/L) under aerobic condition at 37°C. Cell growth was monitored turbidimetrically at 600 nm. All reagents were the purest available grade (Sigma Aldrich, USA), and the culture media were obtained from Oxoid (USA).

**Table:**
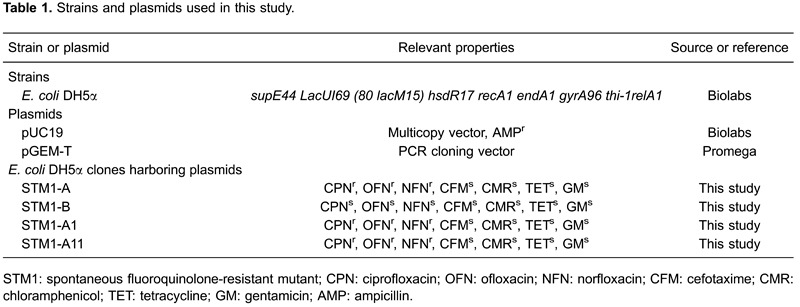

### Recombinant DNA techniques

Unless otherwise indicated, standard protocols were used for isolation of plasmid and chromosomal DNA, bacterial transformation, and western blot analysis (7). Nucleotide sequences were determined by the dideoxy chain termination method (8) with a Terminator Cycle Sequencing kit (Applied Biosystems 3500 Genetic Analyzer, Applied Biosystems, USA). The Basic Local Alignment Search Tool (BLAST) program at the National Center for Biotechnology Information (www.ncbi.nlm.nih.gov/blst.cgi) was used to analyze the sequence data and assess the degree of DNA similarity between the sequences.

### Cloning the ciprofloxacin resistance gene

Chromosomal DNA was prepared from *S. Typhi* STM1 cells by using an Ultra Clean DNA Isolation Kit (MO-BIO, USA). The DNA was partially digested with *Sau*3AI, and fragments of between 1 and 10 kbp were separated by electrophoresis in 0.5% agarose gels and then purified by a Qiaquick Gel Extraction Kit (Qiagen, USA). The DNA fragments were ligated to pUC19 (digested with *Bam*HI and dephosphorylated with bacterial alkaline phosphatase) with T4 DNA ligase (Boehringer Mannheim GmbH., Germany). Competent *Escherichia coli* DH5α cells, transformed with the recombinant plasmids, were spread on LB agar plates containing 0.5 μg/mL of ciprofloxacin and 100 μg/mL of ampicillin. The plates were incubated under aerobic conditions at 37°C for 24 h and the colonies formed were collected. Plasmid-containing transformants were isolated, reintroduced into *E. coli* DH5α cells, and the transformation mixture was spread onto LB agar plates. The plates were incubated at 37°C for 24 h. Plasmids from the transformants were isolated.

### Drug susceptibility tests

The minimum inhibitory concentrations (MICs) of the antimicrobial agents were determined by the broth microdilution technique (9). Using 96-well microtiter plates, two-fold serial dilutions of the antimicrobial agents in 100 μL of antibiotic medium 3 with an inoculum of 1×10^3^ to 1×10^4^ colony forming units (CFUs) per logarithmic-phase cell sample were prepared. The concentration range used was 0.01 to 32 μg/mL. MIC is defined as the lowest concentration of the antimicrobial agent that inhibits visible growth after 18 to 24 h of incubation at 37°C. The MICs reported here represent the mean values of quadruplicate experiments.

### Isolation of ciprofloxacin-resistant mutants

Spontaneous ciprofloxacin-resistant mutants were selected by plating a 0.1 mL sample of an overnight culture of the selected strain in Mueller-Hinton broth (final inoculum 10^7^−10^8^ CFU/mL) onto Mueller-Hinton agar plates containing ciprofloxacin at a concentration of approximately 12× the MIC (0.8 µg/mL) for the selected *S. Typhi*isolate. After 48 h of incubation at 37°C, the grown colonies were counted and streaked onto another plate containing the same concentration of ciprofloxacin to obtain pure colonies of the ciprofloxacin-resistant mutants. The mutation frequency was calculated by dividing the number of resistant cells by the number of viable cells in the original sample.

### Protein analysis of crude bacterial extracts

Crude bacterial cell extracts, prepared as described by Sambrook et al. (7), were analyzed by sodium dodecyl sulfate-polyacrylamide gel electrophoresis (SDS-PAGE). A lower separation gel (10% acrylamide and 0.1% SDS in 0.375 M Tris-HCl buffer, pH 8.8) and upper stacking gel (5.0% acrylamide and 0.1% SDS in 0.125% M Tris-HCl buffer, pH 6.8) were used.

### DNA gyrase assays

DNA gyrase was isolated and purified according to a method described previously (10). Relaxed Bluescript II plasmid substrate DNA (Agilent technologies, USA) was prepared by treatment of the closed circular plasmid DNA with topoisomerase I according to the manufacturer’s recommended protocol.

DNA supercoiling assays were performed as described previously (11). One unit of gyrase is defined as the amount of enzyme required to catalyze the conversion of one-half of a relaxed closed circular DNA to the supercoiled form in 30 min at 37°C in a standard gyrase reaction containing 0.4 μg of DNA.

IC_50_ is defined as the fluoroquinolone concentration that inhibits 50% of the supercoiling activity of gyrase in a standard gyrase reaction. A control reaction without quinolone was included. After staining the gel in 0.5 μg/mL of ethidium bromide, the IC_50_ was determined by visual comparison with the control reaction.

### Western blot analysis of RecA

The level of RecA expression in crude bacterial extracts was determined with rabbit antiserum specific for the RecA protein (7). Equal amounts (30 µg each) of each protein sample were separated by SDS-PAGE on a 10% gel. The gel was then equilibrated in transfer buffer containing 10 mM Tris base, 200 mM glycine, and 10% methanol for 5 min before it was electroblotted onto a polyvinylidene difluoride membrane (Applied Biosystems). Immunocomplexed proteins were detected by alkaline phosphatase-conjugated secondary antibodies and the chromatic substrates nitroblue tetrazolium and 5-bromo-4-chloro-3-indolylphosphate.

## Results

### Isolation of ciprofloxacin-resistant mutants

Three ciprofloxacin-resistant mutants (STM1-STM3) were isolated from the *S. Typhi* isolate. The mutation frequency of the isolated mutants ranged between 10^−8^ and 10^−7^. Therefore, the isolated mutants were mostly obtained by single-step mutation.

### *In-vitro* susceptibility testing

As shown in Table 2, the MICs of the fluoroquinolones tested against the selected mutants increased by 16-63× those of the parent strain. However, for the other antibiotics tested, in comparison with the parental line, no change in the sensitivity of the mutants was noted.

**Table:**
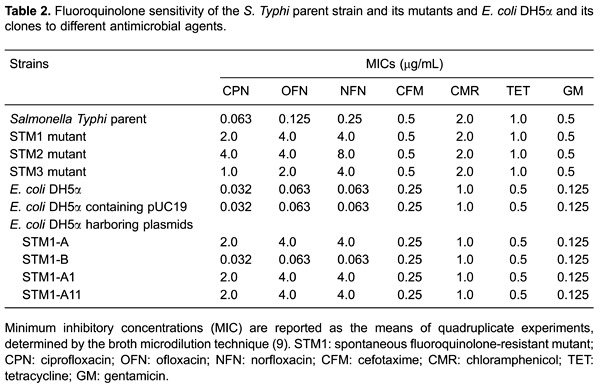

### Inhibition of DNA gyrase supercoiling activity by ciprofloxacin

The inhibitory effect of ciprofloxacin on DNA gyrase supercoiling activity was measured by a supercoiling assay. The results showed that the IC_50_ of ciprofloxacin against the DNA gyrase enzymes isolated from two mutants, STM2 and STM3, increased by 5-10× that of the parent strain. However, no change in the ciprofloxacin sensitivity of the DNA gyrase from the STM1 mutant as compared with that of its parent strain was observed. Therefore, DNA gyrase from the STM1 mutant played no role in the STM1 ciprofloxacin resistance phenotype. Accordingly, the STM1 mutant was selected for further examination to clarify the mechanism of resistance to fluoroquinolones.

### Cloning *S. Typhi* to investigate its genetic determinants of fluoroquinolone resistance

During identification of the genetic determinants of fluoroquinolone resistance in STM1, a shotgun genomic library of this mutant strain was constructed using plasmid pUC19 as the cloning vector. The resulting shotgun library cocktail was introduced into *E. coli* DH5α by transformation, and the transformants were selected on LB agar plates containing 100 μg of ampicillin/mL and 0.5 μg of ciprofloxacin/mL. One colony (STM1-A) was obtained, and the presence of recombinant plasmids in this clone was confirmed by restriction enzyme digestion. The MICs of ciprofloxacin, ofloxacin, norfloxacin, cefotaxime, chloramphenicol, tetracycline, and gentamicin were determined for this clone. As shown in Table 2, the STM1-A-harboring *E. coli* clone only exhibited phenotypic resistance to ciprofloxacin, ofloxacin, and norfloxacin. Further studies were carried out to molecularly characterize this clone.

### Molecular characterization of the STM1-A clone

**Figure 1:**
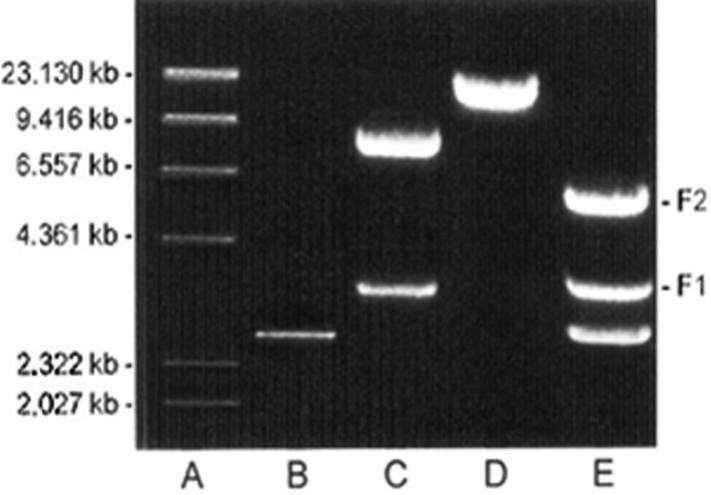
Restriction digest of the 7-kb chromosomal insert from the STM1-A plasmid with *Eco*RI (*C*), *Hind*III (*D*), and *Eco*RI and *Hind*III (*E*) restriction endonucleases. *Hind*III digested λ DNA (*A*); pUC19 alone (*B*).

The 7.0 kb chromosomal insert from STM1-A, digested with *Eco*RI and *Hind*III restriction endonucleases, generated two DNA fragments of approximately 4 and 3 kb (Figure 1). These two DNA fragments were cloned separately into pUC19, thereby forming two plasmids, STM1-A1 and STM1-A2, for subcloning analysis. The MICs of the *E. coli* clones harboring the resultant plasmids (STM1-A1 and STM1-A2) were determined. The results (Table 2) revealed that plasmid STM1-A1 harbored the gene that confers the selective fluoroquinolone resistance phenotype in *E. coli* DH5α. In contrast, the *E. coli* clone harboring the other plasmid (STM1-A2) had the same degree of fluoroquinolone sensitivity as that of *E. coli* DH5α. The nucleotide sequence of the 4 kb insert from STM1-A1 was determined and subsequently analyzed by a homology search against sequences in the non-redundant database of *S. Typhi* at the NCBI with the BLAST program. The results of the sequence comparison indicate that the 4 kb insert of STM1-A1 contains an intact open reading frame that shared identity in the nucleotide sequence of *Salmonella enterica* subsp. *enterica*serovar Typhi str. CT18 with the *recA* gene (Accession No. AL513382.1). Thus, the *recA* gene may play a role in the development of selective resistance against fluoroquinolones.

The *recA* sequence, including the putative ribosomal binding site preceding it, was PCR amplified from STM1-A1. The primers were 5′-CCATGGATGGCTATCGACGAAAAC-3′(*Nco*I forward primer) and 5′-TTCGAATTAAAAATCTTCGTTGG-3′(*Hind*III reverse primer). The PCR products were purified, cloned into a pGEM-T plasmid vector, and introduced into *E. coli* DH5α. The MICs for the *E. coli* clones harboring the resultant plasmid (STM1-A11) were determined. The results (Table 2) revealed that the *E. coli* clone harboring the STM1-A11 plasmid retained its resistance to fluoroquinolones.

The *recA* gene was also PCR amplified from the genomic DNA of wild-type *S. Typhi* and cloned into pGEM-T. Its nucleotide sequence was determined and compared with that of STM1-A11. A 100% homology score between the two nucleotide sequences was obtained.

The DNA gyrase assay results (Table 3) showed the same IC_50_ of ciprofloxacin against the DNA gyrase enzymes isolated from *E. coli* DH5α and the *E. coli* clones harboring the STM1-A1, STM1-A2, and STM1-A11 plasmids. Therefore, DNA gyrase was not involved in the development of selective phenotypic fluoroquinolone resistance among the *E. coli* clones we obtained.

**Table:**
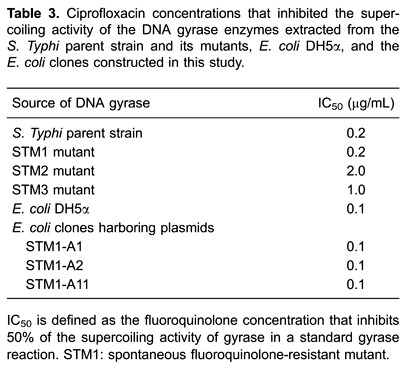

### Protein analysis of crude bacterial extracts

**Figure 2:**
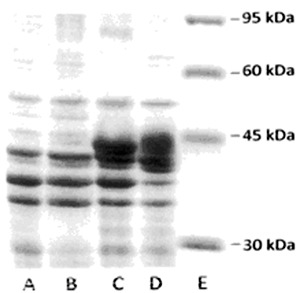
Coomassie brilliant blue stained SDS-PAGE result showing the protein content of crude extracts from samples. *Lane A*: *E coli* DH5α host cells grown in Luria-Bertani broth. *Lanes B-D*: recombinant *E. coli*cells harboring pUC19, STM1-A1, and STM1-A11, respectively. *Lane E*: protein size standard.

The SDS-PAGE analysis showed there was overexpression of proteins of approximately 40 kDa in crude extracts (Figure 2) from the *E. coli* clones harboring STM1-A1 and STM1-A11 plasmids as compared with crude extracts from *E. coli*DH5α. Additionally, where the *E. coli* clones harbored pUC19 plasmids alone, no obvious difference in the protein profile was observed when compared with that of *E. coli* DH5α.

**Figure 3:**
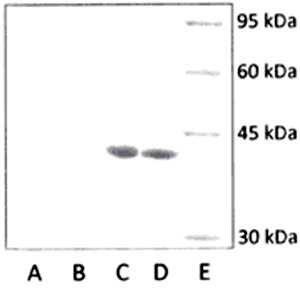
Western blot of RecA protein. *Lane A*: *E coli* DH5α host cells grown in Luria-Bertani broth. *Lanes B-D*: *E coli* recombinant cells harboring pUC19, STM1-A1, and STM1-A11, respectively. *Lane E*: protein size standard.

Western blot analysis using an anti-recA antibody confirmed that the overexpressed 40-kDa protein in the *E. coli* clones harboring STM1-A1 and STM1-A11 was that of the RecA protein (Figure 3).

## Discussion

RecA, a multifunctional protein, is involved in the induction of SOS responses. In response to an SOS-inducing treatment or condition, a signal (involving regions of single-stranded DNA) is generated that stimulates expression of the SOS regulon. RecA protein binding to single-stranded DNA regions in the presence of a nucleoside triphosphate forms a nucleoprotein filament and converts RecA protein to an activated form (12).

Fluoroquinolones induce a certain degree of DNA damage or interference with DNA replication in cells (13). This, in turn, could serve as a signal to activate the coprotease activity of RecA to trigger the SOS regulatory system cascade, resulting in a higher level of resistance to fluoroquinolones. In one study, RecA involvement in fluoroquinolone resistance was inferred by the increase in the sensitivity of RecA mutants to these compounds (5). RecA expression increases the level of resistance to fluoroquinolones in *E. coli* DH5α through its function in the SOS response. RecA protein plays an important role in the coordinated expression of the SOS regulatory system in response to DNA damage. Additionally, there is an absolute requirement for *recA* in all homologous recombination in *E. coli* where it catalyzes synapsis and strand exchange between homologous molecules (14,15). Piddock and Walters (16) studied the bactericidal effects of various fluoroquinolones on different strains of *E. coli* with mutations in genes for the SOS response. They observed that mutants with constitutive RecA expression survived longer than the wild-type *E. coli* strain.

In the present study, an *S. Typhi* STM1 mutant was isolated by a single-step mutation. STM1 was characterized by its selective resistance to fluoroquinolones. The results of a DNA gyrase assay revealed that the DNA gyrase isolated from this mutant played no role in the selective fluoroquinolone resistance phenotype. In addition, the *E. coli* clone that harbored the STM1-A1 plasmid had the same fluoroquinolone resistance phenotype. Sequence analysis indicated that the 4 kb insert in STM1-A11 contained the *recA* gene. When the *recA* gene sequence was PCR amplified from STM1-A11 DNA, and then purified, cloned, and introduced into *E. coli* DH5α, only one of the *E. coli* clones obtained that contained the *recA* gene exhibited selective fluoroquinolone resistance. Accordingly, RecA expression in the *S. Typhi* STM1 mutant in *E. coli* DH5α conferred selective fluoroquinolone resistance. To confirm this finding, the *recA* gene was PCR amplified from the genomic DNA of wild-type *S. Typhi* and then cloned into pGEM-T. The sequence of the inserted region (*recA* gene) was determined and compared with that of STM1-A11. The results showed complete identity between the two nucleotide sequences.

RecA expression in the *E. coli* clones from this study was investigated by comparative protein analysis of the crude extract and crude outer membrane proteins of the clones, and compared with the protein analysis of the wild-type *E. coli* DH5α. The results of the protein analysis showed there was overexpression of RecA protein in crude extracts and crude outer membrane proteins of the *E. coli* clones harboring STM1-A1 and STM1-A11 plasmids, as compared with the expression profile of *E. coli* DH5α. RecA protein expression in *E. coli*clones harboring STM1-A1 or STM1-A11 in samples of crude outer membrane proteins was confirmed by western blot analysis. The increased association of the RecA protein with the membrane fractions that was observed in this study requires the presence of the activated form of RecA and this association may contribute substantively to the SOS response (17,18). Therefore, overexpression of RecA confers resistance to fluoroquinolones in *E. coli* DH5α.

Accordingly, the results suggest that transformation of the *recA*gene of *S. Typhi* into *E. coli* DH5α resulted in RecA protein expression and development of the selective fluoroquinolone resistance phenotype in the *E. coli* DH5α clone.
